# High Levels of Circulating Type II Collagen Degradation Marker (CTx-II) Are Associated with Specific *VDR* Polymorphisms in Patients with Adult Vertebral Osteochondrosis

**DOI:** 10.3390/ijms18102073

**Published:** 2017-09-29

**Authors:** Sabina Cauci, Marco Viganò, Laura de Girolamo, Paola De Luca, Carlotta Perucca Orfei, Giuseppe Banfi, Giovanni Lombardi, Marco Brayda-Bruno, Alessandra Colombini

**Affiliations:** 1 Department of Medicine, University of Udine, 33100 Udine, Italy; sabina.cauci@uniud.it; 2 Orthopaedic Biotechnology Lab, IRCCS Galeazzi Orthopaedic Institute, Via R. Galeazzi 4, 20161 Milan, Italy; marco.vigano@grupposandonato.it (M.V.); laura.degirolamo@grupposandonato.it (L.d.G.); deluca.paola@grupposandonato.it (P.D.L.); carlotta.perucca@grupposandonato.it (C.P.O.); 3 Vita-Salute San Raffaele University, 20132 Milan, Italy; banfi.giuseppe@fondazionesanraffaele.it; 4 Laboratory of Experimental Biochemistry & Molecular Biology, IRCCS Galeazzi Orthopaedic Institute, 20161 Milan, Italy; giovanni.lombardi@grupposandonato.it; 5 Department of Orthopedics and Traumatology, Spine Surgery III-Scoliosis Unit, IRCCS Galeazzi Orthopaedic Institute, 20161 Milan, Italy; marco.brayda@spinecaregroup.it

**Keywords:** adult vertebral osteochondrosis, Italian males, *VDR* polymorphisms, type II collagen

## Abstract

Both vitamin D and collagen have roles in osteocartilaginous homeostasis. We evaluated the association between the circulating 25-hydroxyvitamin D (25(OH)D), type I and II collagen degradation products (CTx-I, and CTx-II), and four vitamin D receptor gene (*VDR*) polymorphisms, in Italian males affected by low back pain (LBP) due to herniation/discopathy and/or vertebral osteochondrosis. FokI, BsmI, ApaI, and TaqI *VDR*-polymorphisms were detected through PCR–restriction fragment length polymorphism (RFLP), and circulating 25(OH)D, CTx-I and CTx-II were measured by immunoassays in 79 patients (of which 26 had osteochondrosis) and 79 age-, sex- and body mass index (BMI)-matched healthy controls. Among all 158 subjects, carriers of *FF* and *Ff* genotypes showed lower 25(OH)D than *ff*, which suggested a higher depletion of vitamin D in *F* allele carriers. Higher CTx-I concentrations were observed in *TT* versus *Tt* among controls, and *Tt* versus *tt* among LBP cases, which suggested a higher bone-cartilaginous catabolism in subjects bearing the *T* allele. Higher CTx-II concentrations were observed in patients with osteochondrosis bearing *FF*, *bb*, *TT*, or *Aa* genotypes in comparison with hernia/discopathy patients and healthy controls. Vertebral osteochondrosis shows peculiar genotypic and biochemical features related to vitamin D and the osteocartilaginous metabolism. Vitamin D has roles in the pathophysiology of osteochondrosis.

## 1. Introduction

A recent Italian cohort study performed on adult patients with low back pain (LBP) confirmed by magnetic resonance imaging (MRI) found that 26.8% (40/149) of male patients and 8.5% (10/118) of female patients had spine osteochondrosis [1,2].

The vertebral osteochondrosis, or Scheuermann disease, belongs to the osteochondrosis group of pathologies, which are characterized by the presence of degenerative necrotic ischemic processes of the ossification nuclei at the growing epiphyses and apophyses beginning in childhood and adolescence [3,4]. Scheuermann disease is the most common cause of structural kyphosis in adolescence. A prevalence of 18–40% of Scheuermann disease radiological signs was reported in the general population, with more males affected [5].

In some cases, especially when the pathology is present at the lumbar level, spine osteochondrosis is asymptomatic, and the related LBP can appear only in adulthood [6,7,8,9].

Apparently, the vertebral osteochondrosis involves defects in the cartilage endplate of vertebrae [10], which is devoted to the vertical growth of the vertebral body. The clinical/radiological manifestations of this pathology are the presence of an irregular vertebral endplate, disc narrowing, subchondral sclerosis, Schmorl’s node (upper or lower disc herniation into the spongious bone of the vertebral body), anterior wedging of the vertebral bodies, and kyphosis [11,12]. However, kyphosis can be present or absent [13].

Morphological studies of spinal osteochondrosis showed the presence of sparse disorganized fibrils in the cartilage matrix, which are likely associated with disturbed collagen synthesis and an abnormal collagen/proteoglycan ratio [11,14,15].

The etiology of osteochondrosis is elusive, and likely multifactorial. Evidence highlights the involvement of an impaired blood supply causing oxygen and nutrition insufficiency at the vertebral ossification nuclei and consequent cell necrosis [4], but mechanical damage/repeated microtrauma can also contribute [8]. Moreover, a genetic influence was observed: a high prevalence of osteochondrosis in male monozygotic twins [16,17] and an inheritance of the autosomal dominant type [18,19] support the genetic etiology hypothesis.

An increasing number of research studies point to vitamin D involvement not only in spinal bone homeostasis, but also in cartilaginous tissues, and consequently in etiology of LBP [20]. Virtually all actions of vitamin D occur by activation of the vitamin D nuclear receptor (VDR), which after translocation into the cell nuclei and by binding to thousands of vitamin D recognition sites (VDREs) can up- and downregulate hundreds of human genes [21]. Notably, VDR is present in osteoblasts [22] and intervertebral disc (IVD) cells [23,24]. Such cells can be found in the osteocartilaginous osteophytes, i.e., neo-formations showing some stages of bone turnover and remodeling. Osteophytes likely represent a healing response of the disc to structural degeneration, which involves interplay between the hyperplasia of bone tissue and the neo-formation of cartilage. In view of the importance of the vitamin D hormone in osteocartilaginous metabolism, the vitamin D endocrine system pathways might have a crucial role in the development of IVD and osteochondrosis pathological features [20]. Of note, polymorphisms in the vitamin D receptor gene (*VDR*) have been showed to be associated with LBP and particularly with spine pathologies involving the IVD, such as herniation and discopathies, or affecting both IVD and endplate, such as osteochondrosis [20,25]. Nevertheless, there is no agreement concerning these associations [26], and there are no functional studies evaluating the real influence of *VDR* genetic variants on IVD pathologies [20].

By analyzing the four most studied *VDR* polymorphisms, namely FokI, BsmI, ApaI, and TaqI in an Italian cohort of LBP patients, a link was observed between the aforementioned genetic variants and specific LBP-associated lumbar spine pathologies [1,2,27]. In particular, peculiar associations were observed between the *FF*, *Aa*, and *bbAaTT* genotypes, the *T* and *F* alleles (but in males only [2]), and the increased risk of developing vertebral osteochondrosis [1,27]. Collagen homeostasis likely has important roles in IVD diseases [28]. Of interest, in a recent study, males with IVD pathologies, particularly those with osteochondrosis, showed higher cross-linked type 2 collagen fragments, called CTx-II (which is considered a marker of cartilage metabolism) than healthy controls [21]. Thus, LBP patients affected by IVD diseases, and particularly those with vertebral osteochondrosis, present peculiar genotypical/biochemical and phenotypical features, which distinguish them from the general LBP group of patients that have spine pathologies, but without osteochondrosis.

In this context, the aim of this study is to evaluate the possible association between the plasma vitamin D concentrations and the circulating levels of type I and II collagens C-telopeptides’ degradation products (CTx-I and CTx-II, respectively), as markers of osteocartilaginous damage, and the presence of specific genotypes/alleles in the *VDR* polymorphic sites in a cohort of Italian males affected by two LBP-related conditions, osteochondrosis and disc herniation/discopathy without osteochondrosis, versus healthy controls. The results of the present study will allow researchers to better understand the molecular basis of the modulation effects of *VDR* genetic variants in disc degeneration-related pathologies.

## 2. Results

### 2.1. Genotypes and Alleles Frequencies in the Study Population

LBP patients (*n* = 79) and healthy controls (*n* = 79) had an age range of 19–62 years (mean ± SD 40.8 ± 9.2 years), and a BMI range of 18.3–37.2 (mean ± SD 25.9 ± 3.8 kg/m^2^); cases and controls did not differ in terms of age and BMI. Among patients, 33 had herniation without discopathy, 20 had discopathy with (*n* = 14) or without (*n* = 6) herniation, and 26 had osteochondrosis with (*n* = 20) or without (*n* = 6) discopathy and herniation.

The frequencies of FokI, BsmI, ApaI, and TaqI *VDR* genotypes and alleles in healthy controls (*n* = 79), LBP cases (*n* = 79) and two different pathological subgroups constituted of LBP patients with hernia/discopathy without osteochondrosis (*n* = 53) and patients with osteochondrosis (*n* = 26) are reported in Table 1. The frequencies of genotypes and alleles of the four *VDR* polymorphisms were consistent with those reported in previously published studies from the same research group [1,2,27]. Statistically significant differences were evaluated by comparing patients groups with healthy controls, and by comparing the two patient subgroups. The *TT* genotype and *T* allele were more frequent in osteochondrosis patients than in the healthy controls (Odds Ratio (OR) = 3.3, 95% Confidence Interval (CI) = 1.3–8.2, *p* = 0.012; OR = 2.7, 95% CI = 1.3–5.6, *p* = 0.009, respectively). An increased tendency for the risk to develop osteochondrosis was observed for *b* allele carriers compared with healthy controls (OR = 1.8, 95% CI = 0.9–3.6, *p* = 0.084). Consequently, the *B* allele tended to be protective for osteochondrosis. No differences were noted between hernia/discopathy and osteochondrosis patients.

Present results concerning the osteochondrosis patients confirmed previously published data in a larger cohort of LBP Italian patients [1,27].

### 2.2. FokI Polymorphism, and 25(OH)D, CTx-I and CTx-II Circulating Concentrations

Concentrations of plasma 25(OH)D, CTx-I and CTx-II were evaluated according to specific genotypes and alleles in all 158 study subjects, and specifically in 79 healthy controls, and in the 79 LBP patients. Additionally, LBP cases were further subdivided in 53 hernia/discopathy patients without osteochondrosis, and in 26 patients with osteochondrosis.

**Figure 1:**
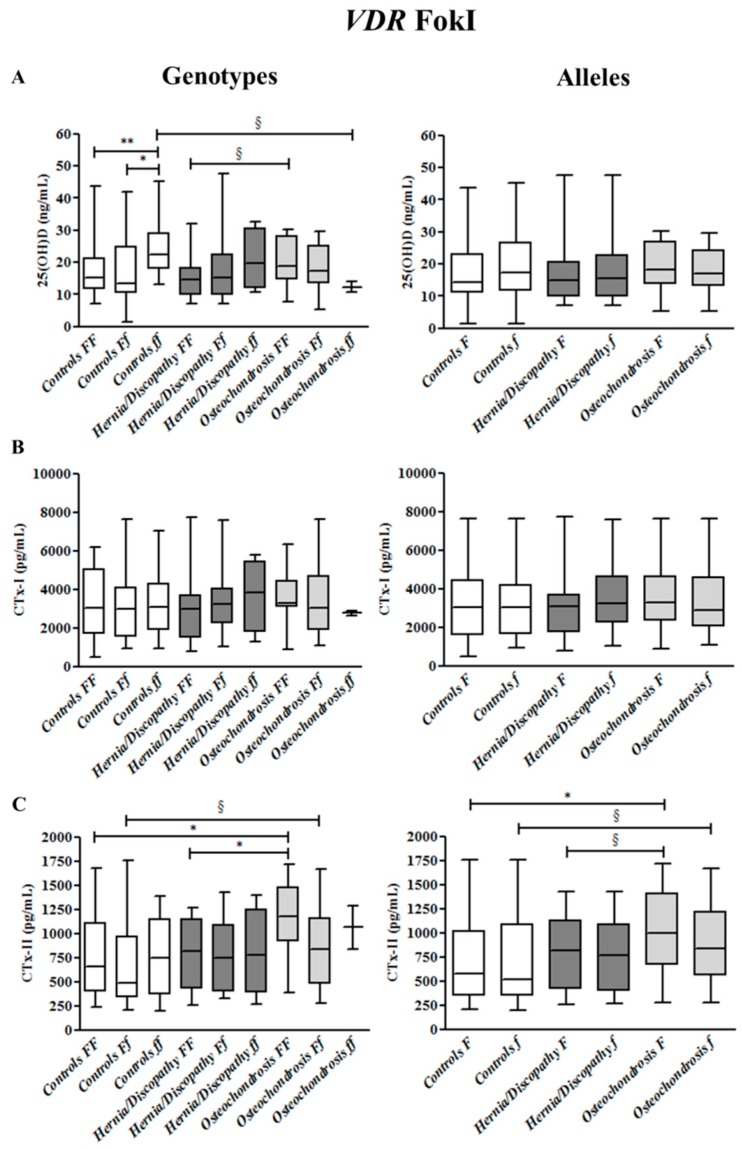
25hydroxyvitamin D (25(OH)D) (**A**), cross-linked C-telopeptides of type I collagen (CTx-I) (**B**), and cross-linked C-telopeptides of type II collagen (CTx-II) (**C**) plasma concentrations in controls and in the subgroups of patients with hernia/discopathy and osteochondrosis, distributed according to FokI *VDR* genotypes/alleles. The box and whisker plots show the median, 25th, and 75th percentile (interquartile) (the box), and minimum and maximum value (the whiskers). * *p* < 0.05. ** *p* < 0.01. ^§^ indicates a tendency.

Median 25(OH)D concentrations were higher in all study subjects that carried the *ff* genotype (median 22.1 ng/mL) than those with *Ff* (median 14.9 ng/mL) and *FF* genotypes (median 15.4 ng/mL) (+32%, *p* = 0.031; and +30%, *p* = 0.007, respectively) (Supplementary Figure S1). However, this result was observed in healthy controls only (*ff* median 22.5 ng/mL versus *Ff* 13.4 ng/mL, +41%, *p* = 0.017 and versus *FF* 15.1 ng/mL, +33%, *p* = 0.004), not in LBP patients (Figure 1).

**Figure 2:**
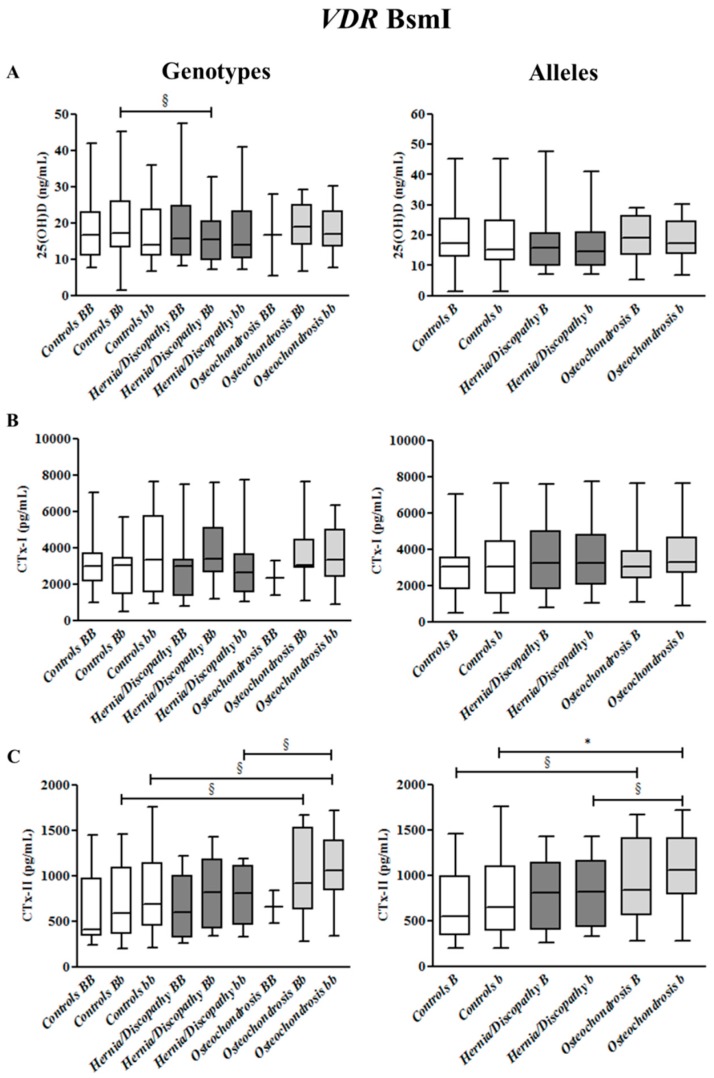
25hydroxyvitamin D (25(OH)D) (**A**), cross-linked C-telopeptides of type I collagen (CTx-I) (**B**), and cross-linked C-telopeptides of type II collagen (CTx-II) (**C**) plasma concentrations in controls and in the subgroups of patients with hernia/discopathy and osteochondrosis, distributed according to BsmI *VDR* genotypes/alleles. The box and whisker plots show the median, 25th and 75th percentile (interquartile) (the box), and minimum and maximum value (the whiskers). * *p* < 0.05. ^§^ indicates a tendency.

**Figure 3:**
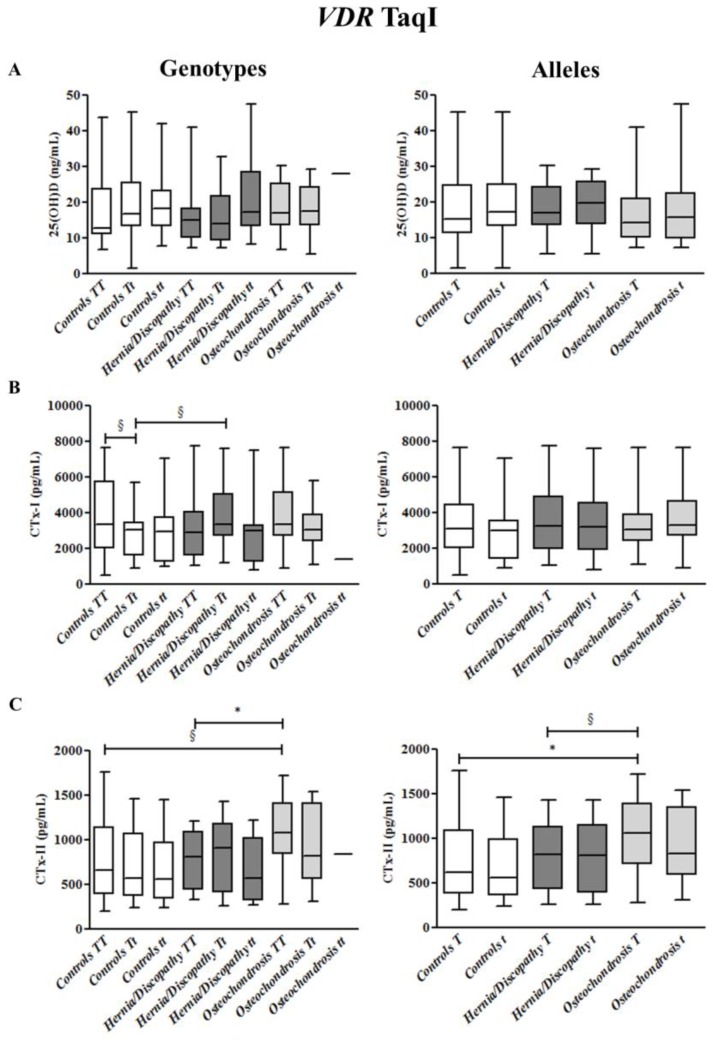
25hydroxyvitamin D (25(OH)D) (**A**), cross-linked C-telopeptides of type I collagen (CTx-I) (**B**), and cross-linked C-telopeptides of type II collagen (CTx-II) (**C**) plasma concentrations in controls and in the subgroups of patients with hernia/discopathy and osteochondrosis, distributed according to TaqI *VDR* genotypes/alleles. The box and whisker plots show the median, 25th, and 75th percentile (interquartile) (the box), and minimum and maximum value (the whiskers). * *p* < 0.05. ^§^ indicates a tendency.

**Figure 4:**
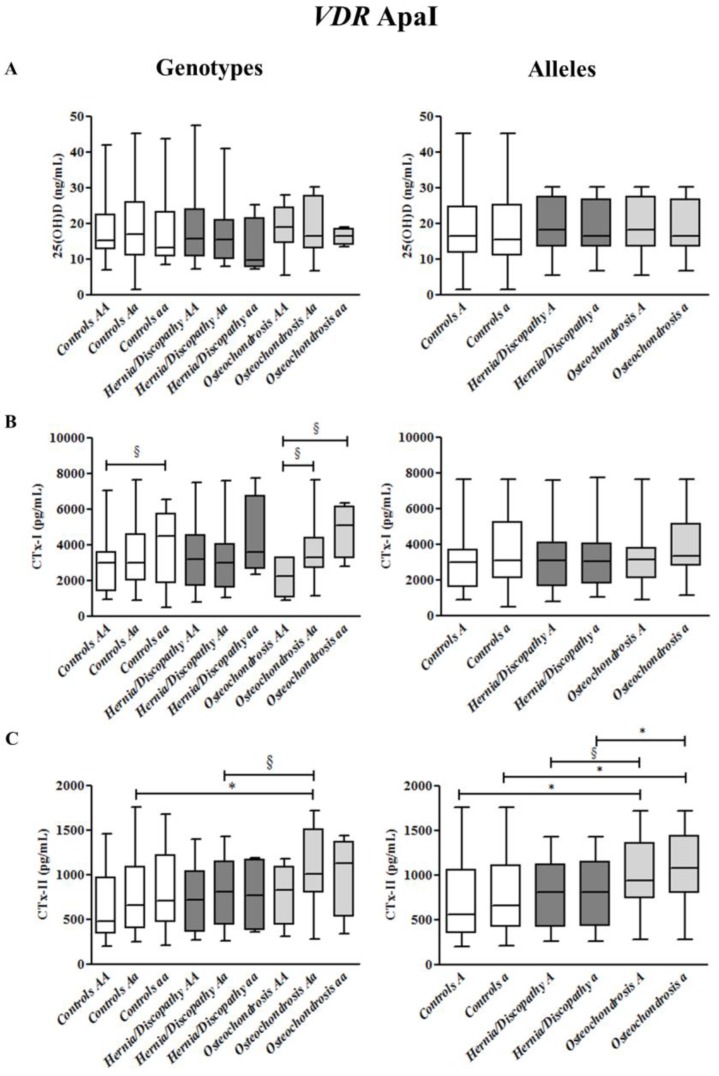
25hydroxyvitamin D (25(OH)D) (**A**), cross-linked C-telopeptides of type I collagen (CTx-I) (**B**), and cross-linked C-telopeptides of type II collagen (CTx-II) (**C**) plasma concentrations in controls and in the subgroups of patients with hernia/discopathy and osteochondrosis, distributed according to ApaI *VDR* genotypes/alleles. The box and whisker plots show the median, 25th and 75th percentile (interquartile) (the box), and minimum and maximum value (the whiskers). * *p* < 0.05. ^§^ indicates a tendency.

At variance, 25(OH)D concentrations did not differ significantly according to BsmI, ApaI, and TaqI genotypes and alleles when comparing all LBP cases versus healthy controls, and hernia/discopathy versus osteochondrosis patients (as shown in Figures S2–S4 and Figure 2, Figure 3 and Figure 4).

CTx-I concentrations did not differ, according to FokI polymorphism (as shown in Figure S1 and Figure 1).

Regarding CTx-II, among *F* allele carriers, higher concentrations were observed in LBP patients (median 883 pg/mL, +34%, *p* = 0.025) than in healthy controls (median 581 pg/mL) (Supplementary Figure S1). However, the increase of CTx-II among *F* carriers was significant in patients with osteochondrosis (median 1000 pg/mL, +42%, *p* = 0.013), but not in hernia/discopathy patients (median 813 pg/mL) compared with healthy controls. *F* carriers with osteochondrosis tended to have higher CTx-II than hernia/discopathy patients (*p* = 0.096). When considering *FF* genotype carriers, higher levels of CTx-II were observed in patients with osteochondrosis (median 1179 pg/mL) than the healthy controls (median 655 pg/mL) (+44%, *p* = 0.017) and hernia/discopathy patients (median 820 pg/mL) (+30%, *p* = 0.013), as illustrated in Figure 1.

### 2.3. BsmI Polymorphism, and CTx-I and CTx-II Circulating Concentrations

BsmI polymorphism did not affect 25(OH)D and CTx-I concentrations; however, some differences were found for CTx-II concentrations (Supplementary Figure S2 and Figure 2).

Among all study subjects (+38%, *p* = 0.017), and among LBP patients (+36%, *p* = 0.037), *bb* genotype carriers showed significantly higher CTx-II levels (median 839 pg/mL and median 947 pg/mL, respectively) compared with *BB* subjects (median 517 pg/mL and 600 pg/mL median, respectively) (Supplementary Figure S2). Among *bb* genotype carriers, a tendency for higher CTx-II was observed in patients with osteochondrosis versus controls (*p* = 0.095) and patients with hernia/discopathy (*p* = 0.069) (Figure 2). Among subjects carrying the *b* allele, increased concentrations of circulating CTx-II were found in LBP patients (median 929 pg/mL, +30%, *p* = 0.027) compared with healthy controls (median 647 pg/mL) (Supplementary Figure S2). Such findings were confirmed for osteochondrosis *b* carriers (median 1056 pg/mL, +39%, *p* = 0.012), but not for hernia/discopathy patients (median 813 pg/mL) when compared with healthy controls (Figure 2).

Moreover, among *b* allele carriers, CTx-II tended to be higher in patients with osteochondrosis than in hernia/discopathy patients (*p* = 0.093).

### 2.4. TaqI Polymorphism, and CTx-I and CTx-II Circulating Concentrations

Concerning CTx-I, slightly higher levels were found in *TT* than *Tt* carriers among controls (*p* = 0.059) (Figure 3) and in *Tt* than *tt* carriers among LBP cases (*p* = 0.086) (Supplementary Figure S3).

Significant increased concentrations of CTx-II were observed among *T* carriers in LBP cases (median 910 pg/mL, +32%, *p* = 0.025) compared with healthy controls (median 620 pg/mL) (Supplementary Figure S3), and in osteochondrosis patients (median 1053 pg/mL, +41%, *p* = 0.013) compared with healthy controls (Figure 3). As illustrated in Figure 3, among *TT* carriers, CTx-II concentrations were higher in patients with osteochondrosis (median 1081 pg/mL, +26%, *p* = 0.041) than in patients with herniation/discopathy (median 803 pg/mL).

### 2.5. ApaI Polymorphism, and CTx-I and CTx-II Circulating Concentrations

Significant increased concentrations of CTx-I were observed among the whole cohort of 158 subjects comparing *aa* (median 4473 pg/mL, +33%, *p* = 0.038) versus *Aa* (median 2983 pg/mL) and *aa* (+32%, *p* = 0.008) versus *AA* (median 3022 pg/mL) genotypes (Supplementary Figure S4). Among LBP cases, this difference was confirmed by comparing *aa* (median 4184 pg/mL, +24%, *p* = 0.042) with *AA* genotype carriers (median 3181 pg/mL) (Supplementary Figure S4).

A tendency for higher CTx-I was observed among healthy controls for *aa* (median 4473 pg/mL) versus *AA* (median 2999 pg/mL) genotypes (*p* = 0.058), and in patients with osteochondrosis for *aa* (median 5090 pg/mL) (*p* = 0.067) and *Aa* (median 3282 pg/mL) (*p* = 0.059) versus *AA* (median 2211 pg/mL) genotypes.

A significant increase of CTx-II was observed in all study subjects upon comparing *Aa* (median 839 pg/mL, +33%, *p* = 0.030) and *AA* (median 562 pg/mL) genotypes. Among *A* allele carriers, CTx-II had higher concentrations in LBP cases (median 838 pg/mL, +34%, *p* = 0.023) than in healthy controls (median 554 pg/mL) (Supplementary Figure S4).

As illustrated in Figure 4, among *a* allele carriers, patients with osteochondrosis (median 1081 pg/mL) had higher CTx-II concentrations than the healthy controls (median 661 pg/mL) (+39%, *p* = 0.015) and the hernia/discopathy patients (median 809 pg/mL) (+25%, *p* = 0.048). However, also among *A* carriers, osteochondrosis patients (median 933 pg/mL, +41%, *p* = 0.014) had higher CTx-II concentrations than healthy controls (median 554 pg/mL).

Finally, among *Aa* genotype carriers, patients with osteochondrosis (median 1003 pg/mL, +35%, *p* = 0.017) showed higher CTx-II levels in comparison with healthy controls (median 654 pg/mL) (Figure 4).

### 2.6. Summary for the Association between CTx-II Circulating Concentrations and VDR Polymorphisms

The differences in CTx-II plasma concentrations in the healthy controls and patients with osteochondrosis according to *VDR* genotypes/alleles are summarized in Table 2. These results are matched with data regarding the association of the same *VDR* polymorphisms in a larger Italian cohort of subjects comprising a total of 50 patients with osteochondrosis (40 males and 10 females) and 252 healthy controls (127 males and 125 females) and including subjects of the current investigation [1,2,27]. The presence of *bb*, *Aa*, and *TT* genotypes, and *F* and *T* alleles, are associated with the risk to develop osteochondrosis in the large cohort. The same genetic traits showed higher CTx-II circulating levels in the present study.

## 3. Discussion

The influence of human DNA polymorphisms on the phenotype and on several biomarkers is intensively studied in order to develop a personalized medical approach to several pathologies [29]. Accumulating evidence points to a nutrigenomic approach and, in this context, vitamin D is increasingly studied [30]. Recently, a number of studies specifically evaluated *VDR* polymorphisms and LBP [25].

In this case control study, a cohort of Italian males affected by two types of lumbar disc degeneration-related spine pathologies was compared with a matched cohort of healthy subjects in term of presence of four specific *VDR* genotypes, blood vitamin D, and the concentration of two catabolic osteocartilaginous markers. The biochemical data were analyzed according to specific *VDR* genetic features to isolate the influence of genetic variants on the development of pathological conditions and systemic biochemical alterations.

Patients with lumbar vertebral osteochondrosis emerged from the current and previous research [1,2,27] as a LBP group of patients with peculiar characteristics. Consistently with frequencies of *VDR* polymorphisms observed in a previously published larger study of Italian LBP patients [27], the *TT* and *Aa* genotypes, and the *T* and *b* alleles were more frequently found in patients with osteochondrosis than in healthy controls. Furthermore, a previous study [21] performed on the same subjects showed that osteochondrosis patients showed higher plasma CTx-II concentrations levels (median 1000 pg/mL) than healthy controls (median 604 pg/mL), and higher levels of this marker in comparison with hernia/discopathies patients without osteochondrosis (median 805 pg/mL), which suggests that osteochondrosis strongly associates with increased cartilaginous catabolism [21].

The main aim of this study was to test the hypothesis of whether specific *VDR* polymorphisms affect the circulating concentrations of vitamin D and markers of collagen degradation.

Concerning plasma 25(OH)D concentrations and *VDR* polymorphisms, our study showed that *ff* carriers had higher 25(OH)D than *FF* and *Ff* carriers among all study subjects and among healthy subjects. Circulating levels of 25(OH)D are the result of the fine tuning of several counteracting mechanisms and are considered to reflect vitamin D body stores [31]. One hypothesis could be that in individuals with the minor *ff* genotype producing a VDR receptor that is three amino acids longer and less active, such balancing requires higher levels of circulating vitamin D [32] in healthy individuals in order to maintain homeostasis.

We found no evidence for *VDR* polymorphisms acting as major modifiers of the association between 25(OH)D concentrations and hernia/discopathy or osteochondrosis. Our data partially concurs with that of Marques Vidigal V et al. [33].

To our knowledge, our study was the first to investigate the relationship between *VDR* polymorphisms and plasma concentrations of CTx-I and CTx-II. The interesting finding regarding the functional FokI *VDR* polymorphism was the observation of higher CTx-II levels in *F* allele carriers osteochondrosis patients compared with healthy controls. Among *FF* carriers, CTx-II reached almost doubled median values in osteochondrosis patients (1179 pg/mL) compared with healthy controls (655 pg/mL), with hernia/discopathy patients showing intermediate values (820 pg/mL). No previous study examined such relationships. We previously showed that vitamin D in vitro inhibits the synthesis of type I and II collagens in the disc cells [24]. It is plausible that among patients with osteochondrosis carriage, who are affected by a higher cartilage catabolism, the *F* allele implies a higher transcriptional activity of the VDR, and results in a pronounced inhibition of resident cells that potentially enhances the development or progression of the pathology.

In our study, CTx-II concentrations were also affected by three silent polymorphisms: BsmI, ApaI, and TaqI, which are located near the 3′ terminus (intron 8/exon 9) of the *VDR*.

The association between CTx-II levels and the *VDR* BsmI polymorphism showed some intriguing results. Among *b* allele carriers, osteochondrosis patients showed almost double the CTx-II of healthy controls. Moreover, among all LBP patients, *bb* carriers had almost double the CTx-II of *BB* carriers. However, the similar trend observed among subgroups of patients was not statistically significant. Further enlarged studies are warranted to assess the role of the *b* allele and the *bb* genotype in promoting type 2 collagen degradation, and consequently increasing the risk for osteochondrosis [27].

A higher cartilage catabolism (as assessed by CTx-II) was also observed in osteochondrosis patients with *T* allele and *TT* genotypes than in the healthy controls and hernia/discopathy patients. These data reinforce the hypothesis that the *T* allele promotes collagen catabolism and consequently osteochondrosis, as already observed in a population-based twin cohort study on Finnish males [34].

Finally, exploring the relationships between ApaI polymorphism and CTx-II, the group with the highest concentrations over 1000 pg/mL were observed in osteochondrosis patients carriers of the *a* allele and *aa* genotype, whereas the lowest concentrations around 500 pg/mL were found in healthy subjects carriers of the *A* allele and *AA* genotype. However, among both *A* and *a* alleles carriers, osteochondrosis patients had higher CTx-II than the healthy controls, which reflects the general CTx-II increase in osteochondrosis patients compared with healthy controls [21].

Furthermore, the *Aa* genotype had higher CTx-II than *AA* carriers among all of the study subjects. It is to note that this heterozygous genotype was already known to be associated with osteochondrosis [27].

In summary, regarding the association between CTx-II and *VDR* polymorphisms in patients with osteochondrosis, we observed the presence of higher CTx-II circulating levels in patients with *bb*, *Aa* ,and *TT* genotypes, and *F* and *T* alleles, in comparison with the healthy controls. These genotypes and alleles were already observed to be associated with the risk to develop osteochondrosis in a larger cohort of Italian subjects [1,2,27].

Concerning CTx-I, which is considered a marker of bone–cartilaginous catabolism, we observed a trend for increased CTx-I levels in *TT* versus *Tt* controls, and *Tt* versus *tt* cases, which suggested an increase in bone catabolism in subjects carrying the *T* allele. The opposite trend was observed for the 25(OH)D, which confirmed the previously reported inverse correlation between the vitamin D hormone and CTx-I levels [21].

Considering *VDR* ApaI polymorphism, the *aa* genotype seemed to be associated with an increased bone catabolism. Of note, a recent immunohistochemical study performed in human melanoma excised cells observed lower VDR protein expression in homozygous *aa* carries [35], which suggests that the *aa* genotype can negatively modulate tissue expression of VDR, and thus affect vitamin D actions. Future studies will be necessary to better assess the role of ApaI polymorphism in collagen degradation and in human diseases in general.

In summary, the associations between the specific *VDR* genetic variants and bone/cartilaginous catabolic markers observed in this study confirm the peculiarity of the vertebral osteochondrosis with respect to other lumbar discopathies.

Since osteochondrosis involves both cartilage and bone metabolism showing particular neoformations such as osteophytes, and since the pleiotropic vitamin D has a role in the metabolic control of both types of these tissues, it is likely that modifications in the hormone’s activity can have a role in the pathophysiology of this condition.

In this context, the only study evaluating the role of vitamin D in the osteochondrosis was performed on a swine model. The authors suggested that the dietary supplementation of 25(OH)D can reduce the development of this condition in pigs through VDR activity enhancement, and speculated that the hormone can reduce the IL-1β and TNF-α and promote TGF-β and IGF-1 release, favoring the cartilage homeostasis [36].

The regulatory effects of VDR on the human genome are much more complex than previously thought [37]. Of interest, a recent genome-wide study by Singh et al. [38] found a genetic link between VDR and NF-kB, a factor that in turn modulates pro-inflammatory cytokines [39].

Our study has some limitations. The main limitation is the limited number of subjects, especially when LBP patients were divided into patient subgroups. The strengths of our study are the well-characterized subgroups of LBP patients and detailed lifestyle, genetic, and biochemical data.

In conclusion, the present study highlighted for the first time that *VDR* polymorphisms can affect blood CTx-I, CTx-II, and vitamin D levels both in LBP patients and healthy subjects. Our findings suggest that further investigations on the genotype-related functional response of the disc cells to vitamin D will be the next steps toward a deeper knowledge of the pathophysiology of disc diseases. Moreover, our results indicate that the focus should be directed to specific subgroups of LBP patients, particularly on those with osteochondrosis, as this condition appears more influenced by the different specific *VDR* polymorphisms.

Very recent research revealed that the influence of variations in DNA sequences on the phenotype is strongly filtered by regulatory networks at higher levels, including cells, tissues, organs, and systems. Thus, understanding better the filtering by regulatory networks at tissue levels may improve our knowledge on physiological and pathological pathways [40].

## 4. Materials and Methods

### 4.1. Subjects and Clinical Evaluation

This protocol named GENODISC01 was approved by the Institutional Ethical Review Board ASL Città di Milano, and this study was conducted in accordance with the Helsinki Declaration (approval date 29 January 2009; amendment for case cohort enlargement and for healthy controls inclusion, approval 12 December 2011).

Seventy-nine white male patients with LBP and lumbar disc herniation, and/or discopathies and/or osteochondrosis, confirmed by magnetic resonance imaging (MRI) performed by a 1.5 Tesla scanner (Avanto, Siemens, Erlangen, Germany, EU) and 79 asymptomatic sex-, age- and BMI-matched healthy subjects were enrolled at the IRCCS Galeazzi Orthopaedic Institute during the European GenoDisc Project, according to the inclusion and exclusion criteria previously described [21]. Briefly, LBP patients and healthy controls filled out a detailed questionnaire comprising clinical and lifestyle data (including weight and height). All study subjects attested that they were not affected by concomitant pathologies such as cervical discopathies, scoliosis, hip, knee and hand osteoarthritis, osteoporosis, fibromyalgia, tumors, lupus erythematosus, and rheumatoid arthritis. Healthy controls had no episodes of LBP longer than one day ever in their life. Diagnosis was performed on the bases of the clinical features by a single senior spine surgeon. Disc herniation was diagnosed in the presence of disc material protrusion/extrusion beyond the posterior margins of the adjacent vertebral bodies, and was often associated with discopathies (degenerative changes of the IVD) and/or osteochondrosis. Osteochondrosis was diagnosed in the presence of disc space narrowing, subchondral sclerosis, wavy endplates, osteophytes, and Schmorl’s node [1].

### 4.2. Samples Collection

Blood samples were collected from the antecubital vein in K_2_EDTA containing tubes (Becton-Dickinson, Franklin Lakes, NJ, USA), centrifuged at 1200× *g* for 10 min at 4 °C, and plasma aliquots and pellet blood cells were stored at −80 °C until assayed.

### 4.3. Analysis of Genotypes

The determination of SNP *VDR*-FokI (C > T), *VDR*-BsmI (G > A), *VDR*-ApaI (T > G), and *VDR*-TaqI (T > C) was performed, as previously described [1,27,41]. Genomic DNA was extracted from blood cells according to the procedure of the DNeasy Midi kit (Qiagen, Duesseldorf, Germany). FokI, BsmI, ApaI, and TaqI polymorphisms of *VDR* were detected through polymerase chain reaction and restriction fragment length polymorphism (PCR–RFLP) methods using appropriate primers, as previously described [1,27]. Capital letters *F*, *B*, *A*, and *T* indicated alleles according to the absence of the restriction site for the FokI, BsmI, ApaI, and TaqI enzymes, respectively, whereas the lower letters *f*, *b*, *a*, and *t* denoted alleles according to the presence of the respective restriction sites. Each *VDR* polymorphism of FokI, BsmI, ApaI, and TaqI was in Hardy–Weinberg equilibrium (HWE). As expected, FokI SNP was not in linkage disequilibrium (LD) with the other three *VDR*-SNPs. BsmI was in LD with ApaI and TaqI, and ApaI was in LD with TaqI [27].

### 4.4. Assessment of Circulating Levels of 25(OH)D, CTx-I and CTx-II

The competitive immunoassay 25-Hydroxy-vitamin D total ELISA (Tecan Group Ltd., Männedorf, Switzerland), cross-linked C-telopeptide fragments of type I collagen (CTx-I) and of type II (CTx-II) collagen ELISAs (D.B.A. Italia s.r.l., Milan, Italy) were used to determine the 25(OH)D (D_2_ and D_3_), CTx-I and CTx-II concentrations in plasma.

All the ELISA kits were used following the manufacturer’s indications.

For 25(OH)D, the lower limit of detection (LoD) was 2.81 ng/mL, while the intra-assay (CVw) and inter-assay (CVb) variations were ≤7.8% and ≤9.2%, respectively.

The lower LoD was less than 53.4 pg/mL for CTx-I and 52.3 pg/mL for CTx-II. Variability CVw and CVb for both the assays were <10% and <12%, respectively.

Differences were considered significant for 25(OH)D when higher than the 6.9%, taking into account the within-person biological variation (CV_W_) [42]. No data concerning the biological variation of CTx-I and CTx-II was found in the literature.

Despite CTx-II being normally measured in urine as the quantification of this marker, it is also possible in serum or plasma [21]. The approved protocol for this study enabled the collection of whole blood in K_2_EDTA-containing tubes, and this accounted for the CTx-II measurement in plasma.

### 4.5. Statistical Analysis

The normality of data distribution was assessed using Kolmogorov–Smirnov and Shapiro–Wilk tests.

Plasma concentration data were not normally distributed, and were reported by box plots indicating the median, 25th and 75th percentile (interquartile, IQR), and minimum and maximum values.

Odds ratios (ORs) and 95% confidence intervals (CIs) were calculated to set the association between alleles or genotypes, and the risk of spine pathologies in LBP cases and controls.

The comparisons of continuous variables were performed by the means of the Student’s *t* and Mann Whitney tests as appropriate. Two-sided significance level was held at 0.05, and *p* values ≤ 0.10 were considered as a tendency to be significant.

Statistical software used was GraphPad Prism version 5.00 (GraphPad software, La Jolla, CA, USA).

## Figures and Tables

**Table 1:** Frequencies of vitamin D receptor (*VDR*) genotypes and alleles in healthy controls, all lower back pain (LBP) cases, and two different pathological LBP subgroups (hernia/discopathy without osteochondrosis and osteochondrosis positive patients).

*VDR*		Healthy Controls	All LBP Cases	Hernia/Discopathy	Osteochondrosis
		*n* = 79 (%)	*n* = 79 (%)	*n* = 53 (%)	*n* = 26 (%)
genotypes	*FF*	32 (40.5)	36 (45.6)	27 (50.9)	9 (34.6)
	*Ff*	34 (43.0)	37 (46.8)	22 (41.5)	15 (57.7)
	*ff*	13 (16.5)	6 (7.6)	4 (7.5)	2 (7.7)
	*BB*	14 (17.7)	14 (17.7)	12 (22.6)	2 (7.7)
	*Bb*	39 (49.4)	36 (45.6)	25 (47.2)	11 (42.3)
	*bb*	26 (32.9)	29 (36.7)	16 (30.2)	13 (50.0)
	*TT*	**26 (32.9)**	38 (48.1)	22 (41.5)	**16 (61.5)**
	*Tt*	40 (50.6)	31 (39.2)	22 (41.5)	9 (34.6)
	*tt*	13 (16.5)	10 (12.7)	9 (17.0)	1 (3.8)
	*AA*	32 (40.5)	25 (31.7)	19 (35.8)	6 (23.1)
	*Aa*	34 (43.0) ^	46 (58.2)	30 (56.6)	16 (61.5) ^
	*aa*	13 (16.5)	8 (10.1)	4 (7.5)	4 (15.3)
alleles	*F*	98/158 (62.0)	109/158 (69.0)	76/106 (71.7)	33/52 (63.5)
	*f*	60/158 (38.0)	49/158 (31.0)	30/106 (28.3)	19/52 (36.5)
	*B*	67/158 (42.4) ^	64/158 (40.5)	49/101 (48.5)	15/52 (28.8) ^
	*b*	91/158 (57.6) ^	94/158 (59.5)	52/101 (51.5)	37/52 (71.2) ^
	*T*	**92/158 (58.2)**	107/158 (67.7)	66/106 (62.3)	**41/52 (78.8)**
	*t*	**66/158 (41.2)**	51/158 (32.3)	40/106 (37.7)	**11/52 (21.2)**
	*A*	98/158 (62.0)	96/158 (60.8)	68/106 (64.1)	28/52 (53.8)
	*a*	60/158 (38.0)	62/158 (39.2)	38/106 (35.9)	24/52 (46.2)

Significant differences were indicated in bold, ^ indicated a tendency by comparing LBP patients versus healthy controls.

**Table 2:** Differences in cross-linked C-telopeptides of type II collagen circulating concentrations between patients with osteochondrosis and healthy controls, according to *VDR* genotypes/alleles.

*VDR* Polymorphisms	Genotypes			Alleles	
FokI	*FF* *	*Ff* ^§^	*ff* ^#^	***F*** *^,^^	*f* ^§^
BsmI	*BB*	*Bb* ^§^	***bb*** ^§,^^	*B* ^§^	*b* *
TaqI	***TT*** ^§,^^	*Tt*	*tt* ^#^	***T*** *^,^^	*t* ^#^
ApaI	*AA* ^#^	***Aa*** *^,^^	*aa*	*A* *	*a* *

* *p* < 0.05 significant higher levels of plasma CTx-II in osteochondrosis patients, ^§^ tendency of higher levels of plasma CTx-II in osteochondrosis patients. ^ risky and ^#^ protective genotypes and alleles observed in a larger cohort of Italian subjects [1,2,27] composed by 50 patients with osteochondrosis (with or without hernia or discopathy) and 252 healthy controls. The genotypes and alleles that are significantly relevant for patients with osteochondrosis compared with the healthy controls are indicated in bold.

## Supplementary Materials

Supplementary materials can be found at www.mdpi.com/1422-0067/18/10/2073/s1.
