# Supplementary material for: High Levels of Circulating Type II Collagen Degradation Marker (CTx-II) Are Associated with Specific VDR Polymorphisms in Patients with Adult Vertebral Osteochondrosis
Source: Int J Mol Sci. 2017 Sep 29;18(10):2073. doi: 10.3390/ijms18102073 (PMC5666755; doi:10.3390/ijms18102073)
Supplement: Supplementary file 1 [file ijms-18-02073-s001.pdf]

Supplementary materials: High Levels of Circulating Type II Collagen Degradation Marker (CTx-II) are Associated with Specific *VDR* Polymorphisms in Patients with Adult Vertebral Osteochondrosis

Sabina Cauci, Marco Viganò [2], Laura de Girolamo, Paola De Luca, Carlotta Perucca Orfei, Giuseppe Banfi, Giovanni Lombardi, Marco Brayda-Bruno and Alessandra Colombini

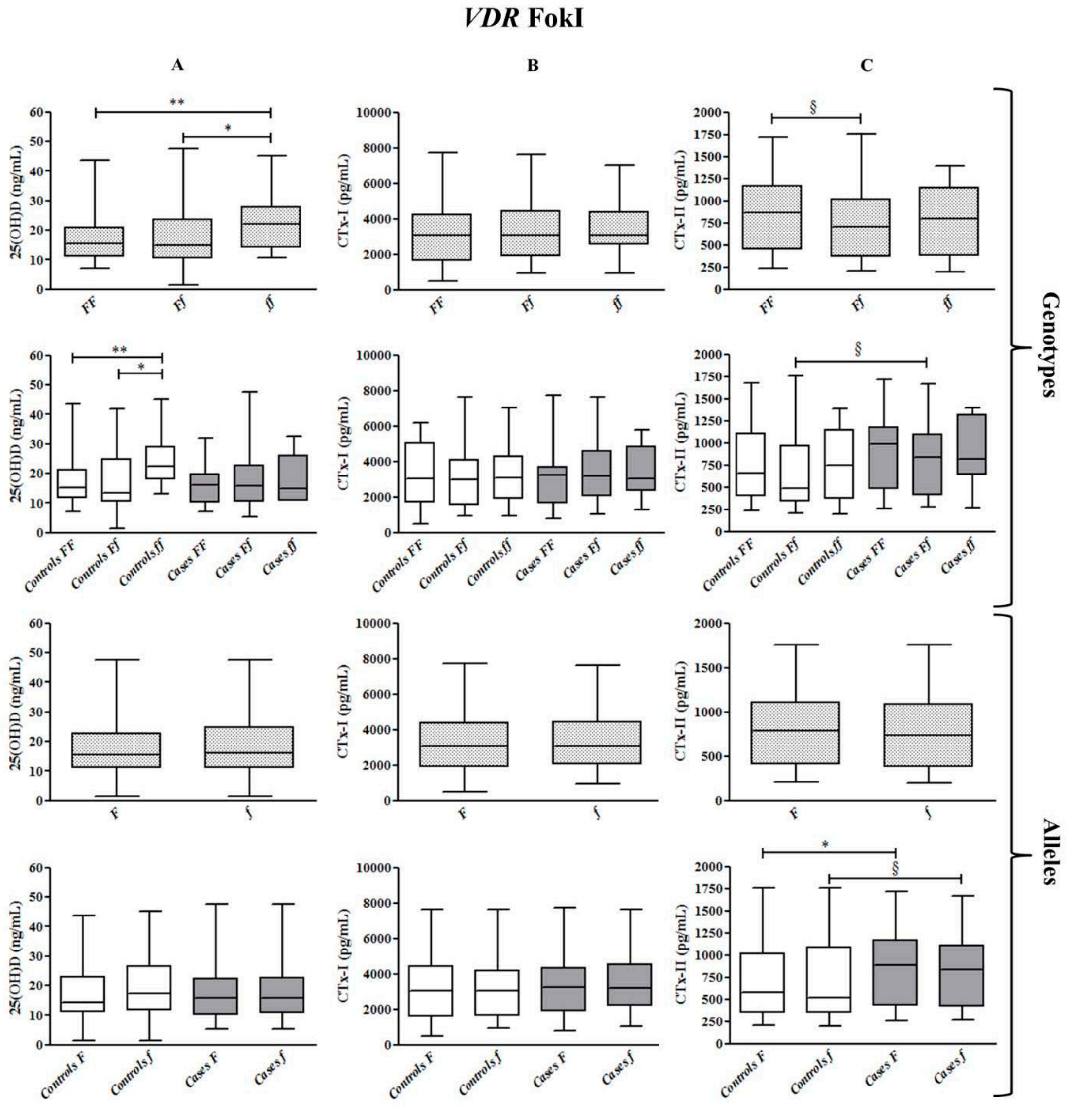

Figure S1. 25hydroxyvitamin D (25(OH)D) (A), cross-linked C-telopeptides of type I collagen (CTx-I) (B) and cross-linked C-telopeptides of type II collagen (CTx-II) (C) plasma concentrations in all subjects (light grey plots) and in all controls (white plots) and all cases (deep grey plots), distributed according to FokI *VDR* genotypes/alleles. The box and whisker plots show the median, 25th and 75th percentile (interquartile) (the box) and minimum and maximum value (the whiskers). * $p < 0.05$. ** $p < 0.01$. § indicates a tendency.

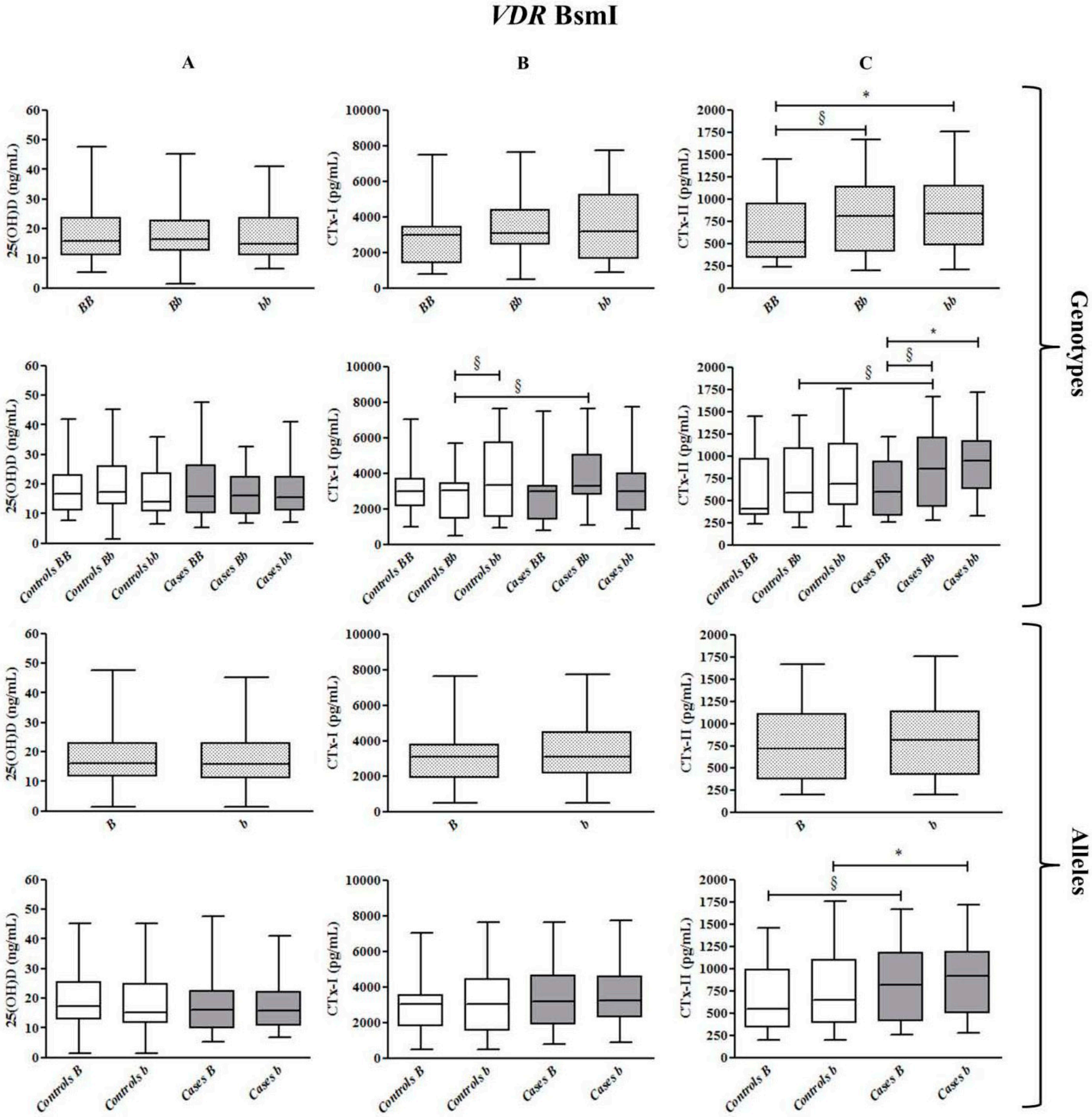

Figure S2. 25hydroxyvitamin D (25(OH)D) (A), cross-linked C-telopeptides of type I collagen (CTx-I) (B) and cross-linked C-telopeptides of type II collagen (CTx-II) (C) plasma concentrations in all subjects (light grey plots) and in all controls (white plots) and all cases (deep grey plots), distributed according to BsmI *VDR* genotypes/alleles. The box and whisker plots show the median, 25th and 75th percentile (interquartile) (the box) and minimum and maximum value (the whiskers). * $p < 0.05$. [§] indicates a tendency.

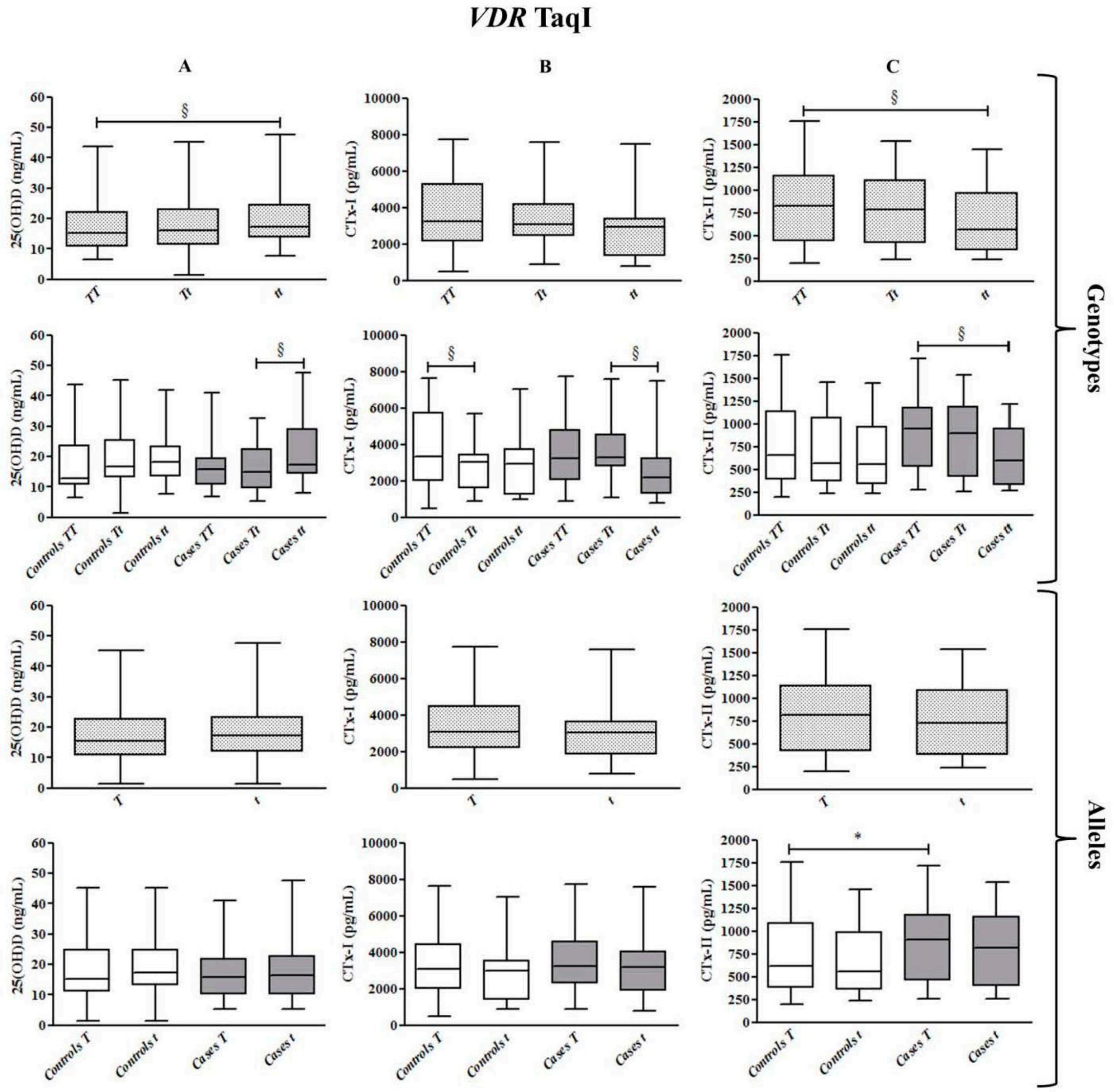

Figure S3. 25hydroxyvitamin D (25(OH)D) (A), cross-linked C-telopeptides of type I collagen (CTx-I) (B) and cross-linked C-telopeptides of type II collagen (CTx-II) (C) plasma concentrations in all subjects (light grey plots) and in all controls (white plots) and all cases (deep grey plots), distributed according to TaqI *VDR* genotypes/alleles. The box and whisker plots show the median, 25th and 75th percentile (interquartile) (the box) and minimum and maximum value (the whiskers). * $p < 0.05$. § indicates a tendency.

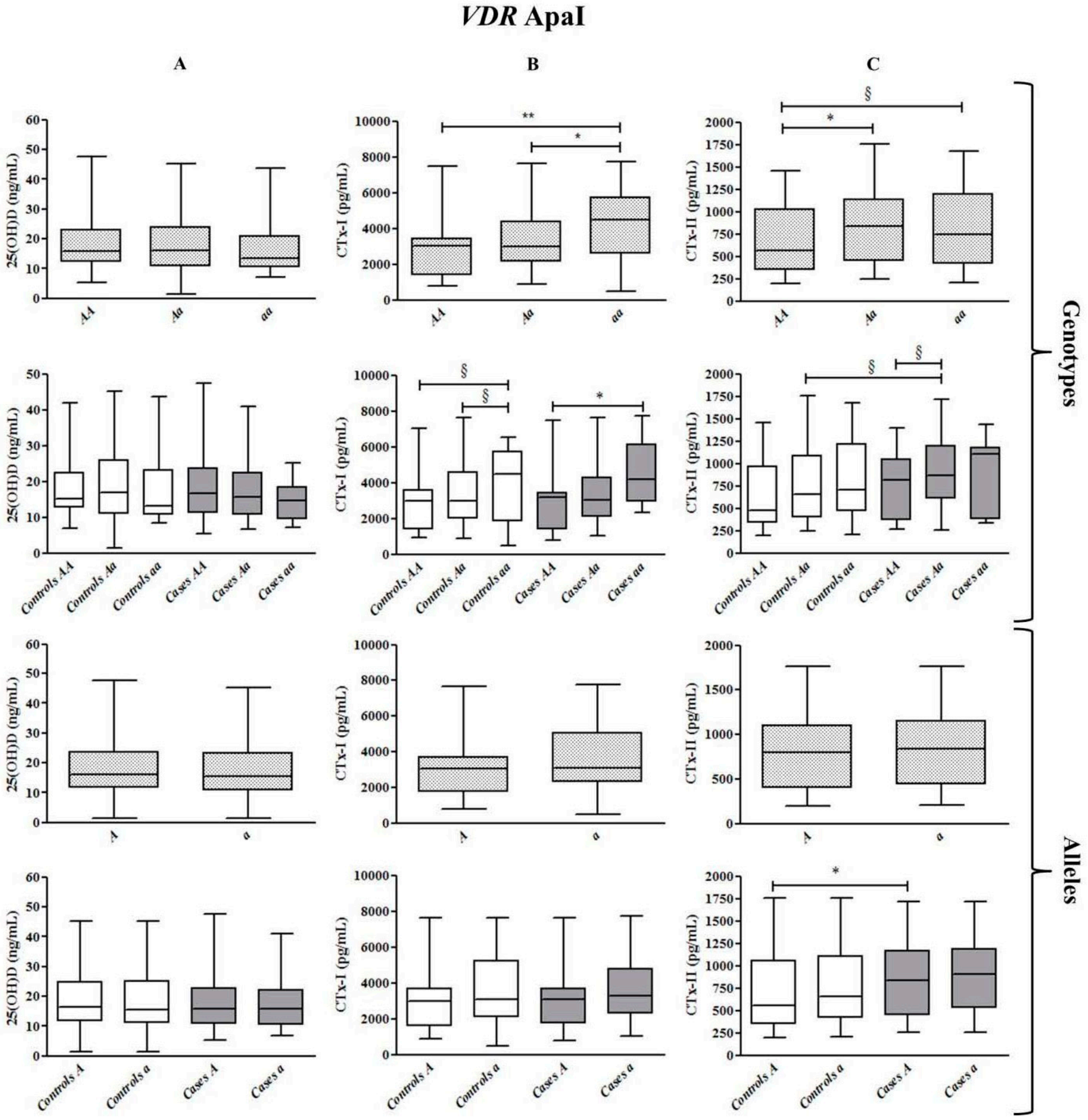

Figure S4. 25hydroxyvitamin D (25(OH)D) (A), cross-linked C-telopeptides of type I collagen (CTx-I) (B) and cross-linked C-telopeptides of type II collagen (CTx-II) (C) plasma concentrations in all subjects (light grey plots) and in all controls (white plots) and all cases (deep grey plots), distributed according to ApaI *VDR* genotypes/alleles. The box and whisker plots show the median, 25th and 75th percentile (interquartile) (the box) and minimum and maximum value (the whiskers). * $p < 0.05$. ** $p < 0.01$. § indicates a tendency.
